# Kinetic response of wild and mutant core codon 70 strains of HCV genotype 1b to pegylated interferon-α and ribavirin therapy

**DOI:** 10.1186/s12985-015-0451-9

**Published:** 2015-12-18

**Authors:** Zhongjie Hu, Ying Liu, Lixia Qiu, Zuopeng Fan, Wei Nie, Shan Liang, Ronghua Jin

**Affiliations:** Department of Hepatitis C & Toxic liver diseases, Beijing Youan Hospital, Capital Medical University, Beijing, the People’s Republic of China; Management center of medical record, Beijing Youan Hospital, Capital Medical University, Beijing, the People’s Republic of China; Beijing Youan Hospital, Capital Medical University, No. 8 Xitoutiao, Youanmenwai, Fengtai District Beijing, 100069 the People’s Republic of China

**Keywords:** Hepatitis C virus, Genotype 1b, Amino acid 70 substitution, Interferon, Viral kinetics

## Abstract

**Background:**

Amino acid (aa) 70 substitution (R70Q/H) in the core protein of hepatitis C virus (HCV) genotype 1b has been shown to be one of the key factors in determining resistance for pegylated interferon-α plus ribavirin combination therapy (PEG-IFNα/RBV). But the exact mechanisms remain unclear. The aim of this study was to investigate the dynamic response of wild and mutant core codon 70 strains to PEG-IFNα/RBV treatment.

**Methods:**

One hundred twelve Chinese patients with chronic HCV 1b infection were enrolled and received a standard protocol of 48 weeks of PEG-IFNα/RBV therapy and 24 consecutive weeks of follow-up. Serial blood samples were obtained at pretreatment baseline, and again at weeks 2, 4, 8, 12, and 24 during therapy for the quantification of 70R and 70Q/H strains. Dynamic characteristics and association with early virological response (EVR), sustained virological response (SVR) and IL28B genotypes were analyzed.

**Results:**

Of the 112 patients enrolled in this study, 93.8 % (105/112) were infected with mixture of 70R and 70Q/H strains before treatment. The 70Q/H strain was dominant in 20.5 % of patients. 42.9 % of patients with dominant 70Q/H exhibited EVR versus 88.6 % of patients with dominant 70R (*P* < 0.001). Furthermore, 35.0 % of patients with dominant 70Q/H exhibited SVR versus 77.4 % with dominant 70R (*P* < 0.001). However, regardless of the dominant strain, virological response types or the IL28B SNP genotypes, 70Q/H strains always exhibited the same response to treatment as the 70R strains and the percentage of HCV harboring the 70Q/H substitution did not change significantly during treatment.

**Conclusions:**

Although the ratio of 70Q/H to 70R is related to the virological response, 70Q/H strains always exhibited the same response as the 70R strains during PEG-IFNα/RBV treatment. Substitution of R70Q/H alone is not enough to lead to resistance to therapy. Positive selection for 70Q/H induced by IFNα was not observed.

## Background

Hepatitis C virus (HCV) infection is one of the major causes of chronic hepatitis and is a global health problem that affects more than 170 million people. In many countries, hepatitis C is the leading cause of cirrhosis and hepatocellular carcinoma (HCC), as well as the leading indication for liver transplantation [1–6]. Of the currently available treatments for chronic HCV infection in China, the most effective is the combination of pegylated interferon alfa (PEG-IFNα) and ribavirin (RBV), which represents a standard treatment approach. However, the long term response to therapy is not satisfactory, especially in patients with HCV genotype 1b, even when administered in a triple therapy regimen available in several countries that includes direct-acting antivirals (DAAs) for the treatment of HCV (telaprevir or boceprevir).

Several factors have been determined to be associated with the failure to respond to PEG-IFNα/RBV therapy and post-treatment relapse, including interleukin 28B single nucleotide polymorphisms (IL28B SNPs), gender, race, age, obesity [7–11], HCV genotypes, viral load, amino acid substitution in the CORE region, and a number of mutations within the NS5A region [12–14]. Of these, substitution of arginine (Arg, R) by glutamine (Gln, Q) or histamine (His, H) at amino acid 70 (R70Q/H) in the core protein and the IL28B polymorphism have been shown to be key factors in determining resistance for both PEG-IFNα/RBV or telaprevir/PEG-IFNα/RVB treatment [15–19]. For patients with the IL28B rs8099917 genotype non-TT, only 12 % of those with 70Q/H exhibited SVR, while 50 % of those with 70R developed SVR [20]. However, the mechanisms that mediate this association remain unclear.

Although most of these studies were performed in Japanese patients, the geographical distribution of genotype 1b strains with core region R70Q/H and IL28B SNPs may be substantially different. The influence of these factors on the response to PEG-IFNα/RVB treatment might be different. In China, about 40 million people are HCV carriers, most of whom are infected with HCV genotype 1b.

Previously, we developed a novel detection system to quantify the virus strains as wild-type aa70 (70R) or mutant (70Q/H) [21]. The present study prospectively enrolled 112 Chinese adults with chronic hepatitis C of genotype 1b who received combination therapy with PEG-IFNα/RVB, and the dynamic changes in 70R and 70Q/H strains during treatment were determined. The aim of this study was to investigate: (1) the distribution of codon 70 in Chinese patients with HCV 1b infection, (2) whether 70Q/H strains are independently resistant to treatment, and (3) whether substitution of amino acid 70 resulted from selection pressure produced by PEG-IFN/RBV treatment.

## Results

### Baseline characteristics

Table 1 summarizes the profiles and laboratory data of the patients at the commencement of antiviral treatment. The patient pool was comprised of 64 males and 48 females, aged 21–75 years (mean, 45.2 years). At baseline, the mean alanine aminotransferase (ALT) and aspartate aminotransferase (AST) levels were 57.3 IU/L (range, 13.9–165.5 IU/L) and 39.8 IU/L (range, 22.6–109.0 IU/L), respectively. The mean leukocyte counts, platelet counts, and hemoglobin levels were 4.6 × 10^9^/L (range, 2.9–9.1 × 10^9^/L), 151.3 × 10^9^/L (range, 41–329 × 10^9^/L), and 134.5 g/L (range, 77–168 g/L), respectively. The mean viremia level was 6.3 log10IU/mL (range, 5.0–7.9 log10IU/mL). There were no significantly difference between the patients with 70R dominance and 70Q/H dominance. However, the rs12979860 CC genotype was associated with wild type aa70 (83.7 % vs. 60 %; *P* = 0.038), which was in agreement with a previous report [17].

**Table 1:** Profile of study patients and baseline clinical features

Characteristic	70R	70Q/H	P
Demographic data			
Number	89	23	
Gender (M/F)	52/37	12/11	0.589^a^
Age (years)	44.8 (8.9)	46.9 (11.0)	0.344^b^
History of blood transfusion^c^	40 (44.9 %)	12 (52.2 %)	0.535^a^
Laboratory data			
Serum alanine aminotransferase (IU/L)	57.5 (24.6)	56.5 (20)	0.867^b^
Serum aspartate aminotransferase (IU/L)	40.3 (16.9)	37.9 (16.0)	0.540^b^
Total serum bilirubin (μmol/L)	17.0 (9.1)	18.6 (12.1)	0.476^b^
Serum albumin (g/L)	39.6 (5.7)	38.0 (6.1)	0.241^b^
γ-Glutamyl transpeptidase (IU/L)	41.9 (18.0)	40.3 (21.4)	0.725^b^
Triglycerides (mmol/L)	1.1 (0.5)	1.0 (0.49)	0.427^b^
Total cholesterol (mmol/L)	4.4 (0.57)	4.4 (0.71)	0.934^b^
Prothrombin time (INR)	1.01 (0.18)	1.04 (0.24)	0.495^b^
Prothrombin activity (%)^d^	91.4 (15.9)	86.3 (16.7)	0.178^b^
Leukocyte count (10^9^/L)	4.75 (1.48)	4.17 (1.0)	0.082^b^
Platelet count ( 10^9^/L)	154.2 (66.2)	140.2 (72.4)	0.379^b^
Hemoglobin (g/L)	135.7 (17.1)	129.8 (22.8)	0.172^b^
HCV RNA level (log10 IU/mL)	6.31 (0.66)	6.28 (0.86)	0.873^b^
IL28B genotype			
rs12979860	77CC/12CT	15CC/8CT	0.038^e^
rs8099917	78TT/11TG	15TT/8TG	0.025^e^

Normal reference ranges: 5–40 IU/L for alanine aminotransferase; 8–40 IU/L for aspartate aminotransferase; 5–20 μmol/L for total serum bilirubin; 36–55 g/L for serum albumin; 10–50 IU/L for γ-Glutamyl transpeptidase; 0.22–1.69 mmol/L for triglycerides; 3.9–5.7 mmol/L for cholesterol; 0.8–1.5 INR for prothrombin time; 80–120 % for prothrombin activity; 4–10 × 10^9^/L for leukocyte count; 100–300 × 10^9^/L for platelet count; 110–160 g/L for hemoglobin

^a^Pearson Chi-Square Test

^b^Independent-Samples T Test

Data are the mean (standard deviation) values, except those denoted by ^c^, which represent the number (percentage) of patients

^d^Prothrombin activity (PTA) was calculated by the patient prothrombin time (PPT) and control prothrombin time (CPT) according to the following formula: PTA = [CPT-(CPT × 0.6)]/[PPT-(CPT × 0.6)] × 100 %

^e^Continuity Correction Chi-Square Test

### Virological responses and adherence

A total of 112 Chinese patients were enrolled in this study. Eight patients discontinued therapy due to adverse effects. Three of the patients (cessation at 4, 5 and 7 weeks) failed to complete follow-up after cessation of therapy. Three patients (cessation at 15, 18 and 24 weeks) achieved complete EVR, but failed to complete follow-up. The other two patients achieved complete EVR, but experienced relapse after cessation of therapy at 14 and 19 weeks respectively. The remaining 104 patients completed all 48 weeks of combination therapy and 24 consecutive weeks of follow-up. All 104 patients received treatment for the expected time and adherence to both drugs was >80 % overall. Among the 109 patients who completed at least 12 weeks of therapy, 79.8 % (87/109) patients exhibited EVR, 21.2 % (22/104) patients failed to respond to the treatment (NVR), 69.2 % (72/104) of the patients achieved SVR.

### IL28B SNP genotypes

The genotypes of two IL28B SNPs (rs12979860 and rs8099917) were measured for each patient. The result shows that these SNPs were in complete linkage disequilibrium and could be nearly interchangeable. Only one patient showed an intermediate haplotype consisting of the unfavourable genotype for rs12979860 (CT) but a favourable genotype for rs8099917 (TT) and the patient failed to follow-up at 15 weeks. Therefore, we selected the rs12979860 as the tag SNP in the studies. Among the 104 patients who completed the 48 weeks of combination therapy and 24 weeks of follow-up, the favourable genotype (CC) was identified in 85 patients (81.7 %), and 66 of them (77.7 %) achieved SVR. The remaining 19 patients were CT heterozygous, only 6(31.6 %) of them achieved SVR. No patient carried the TT genotype in this study set. Conversely, patients with the unfavourable genotype (CT) were significantly more likely to show an NVR (47.4 % vs 15.3 %, *P* = 0.004) (Table 2).

**Table 2:** Virological response by IL28B genotypes and HCV core protein aa70 substitutions

rs12979860	Core 70	EVR	P	SVR	*P*	NVR	P
CC		76/89 (85.4 %)	0.005^a^	66/85 (77.7 %)	<0.001^b^	13/85 (15.3 %)	0.004^a^
CT		11/20 (55.0 %)		6/19 (31.6 %)		9/19 (47.4 %)	
	Wild type	78/88 (88.6 %)	<0.001^a^	65/84 (77.4 %)	<0.001^b^	10/84 (11.9 %)	<0.001^a^
	Mutant	9/21 (42.9 %)		7/20 (35.0 %)		12/20 (60.0 %)	
CC	Wild type	69/76 (90.8 %)	0.003^a^	61/73 (83. 6 %)	0.004^a^	7/73 (9.6 %)	0.002^a^
	Mutant	7/13 (53.9 %)		5/12 (41.7 %)		6/12 (50.0 %)	
CT	Wild type	9/12 (75.0 %)	0.065^a^	4/11 (36.4 %)	1.000^a^	3/11 (27.3 %)	0.07^a^
	Mutant	2/8 (25.0 %)		2/8 (25.0 %)		6/8 (75.0 %)	

*EVR* early virological response; *SVR* sustained virological response; *NVR* non-virological response

^a^Fisher’s Exact Test

^b^Pearson Chi-Square Test

### Prevalence of substitutions of amino acid 70 at baseline in treatment-naive Chinese patients with HCV 1b

**Fig. 1:**
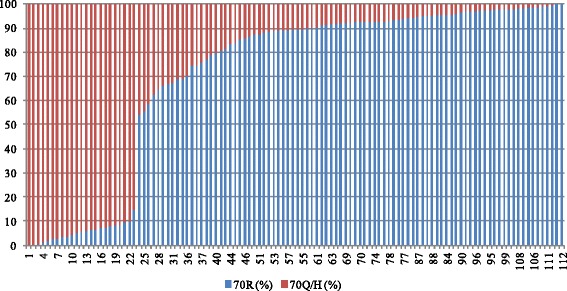
Percentage of 70R and 70Q/H strains in each patient before therapy

The viral loads of HCV with 70R and the 70Q/H substitution were quantified at baseline in all 112 cases. As shown in Fig. 1, 93.8 % (105/112) of the patients were infected with a mixture of 70R and 70Q/H strains before treatment, with the 70Q/H strain dominant in 20.5 % (23/112) patients (Fig. 1).

### Amino acid 70 substitutions at baseline were associated with the virological response to PEG-IFNα/RBV treatment

Analysis of the relationship between the dominant strains (70R or 70Q/H >50 %) and EVR showed that 42.9 % (9/21) of patients in whom 70Q/H was the dominant strain achieved EVR compared to 88.6 % (78/88) of patients with 70R dominance (*P* < 0.001). Furthermore, 35.0 % (7/20) of patients with 70Q/H dominance exhibited SVR, while 77.4 % (65/84) with 70R dominance exhibited SVR (*P* < 0.001). Similar results were found in patients with favourable genotype for rs12979860 (CC). But for patients with unfavourable genotype (CT), although the rates of EVR and SVR in 70R group were higher than that in 70Q/H group, the different was not significant due to the number of cases was too small (Table 2).

### Dynamic changes in 70R and 70Q/H during treatment

**Fig. 2:**
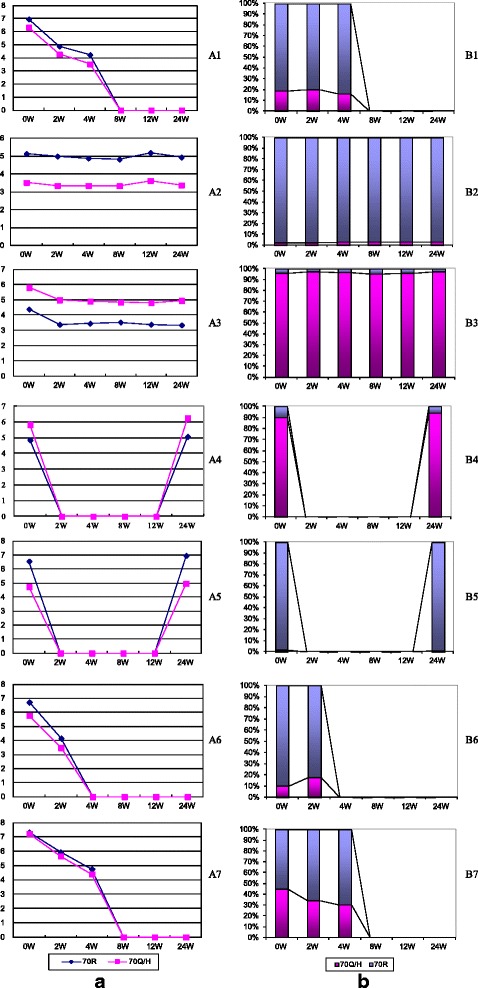
Dynamic changes in 70R and 70Q/H levels before and during PEG-IFNα/RBV treatment. **a**. Viral loads of 70R and 70Q/H strains (Log10 copies/mL). **b**. Percentage of 70R and 70Q/H strains (%). A1/B1: Patient 42 with dominant 70R achieved EVR (rs12979860 CC genotype). A2/B2: Patient 99 with dominant 70R had no response to treatment (rs12979860 CT genotype). A3/B3: Patient 9 with dominant 70Q/H had no response to treatment (rs12979860 CC genotype). A4/B4: Patient 21 with dominant 70Q/H achieved EVR but experienced breakthrough during treatment (rs12979860 CC genotype). A5/B5: Patient 106 with dominant 70R achieved EVR but discontinued therapy due to adverse events at weeks 19 and relapsed quickly (rs12979860 CC genotype). A6/B6: Patient 60 with dominant 70R achieved EVR (rs12979860 CC genotype). A7/B7: Patient 25 with dominant 70R achieved EVR (rs12979860 CT genotype)

Using the serial serum samples collected at weeks 2, 4, 8, 12, and 24 during PEG-IFNα/RBV treatment, the viral loads of HCV with 70R and the 70Q/H substitution were quantified to assess their dynamic response to treatment. The results are summarized in Figure 2. Most notably, no matter the dominant strain or virological response type, the 70Q/H strain always exhibited the same response to treatment as the 70R strain. In patients that exhibited a good response to treatment, the 70Q/H viral load decreased concurrent with that of the 70R strain (A1). If the patients failed to respond to treatment, the viral loads of both the 70R and 70Q/H strains showed little change (A2-3). Furthermore, the percentage of HCV harboring the 70Q/H substitution did not change significantly during treatment (B1-3), even in patients that experienced virological breakthrough (B4) or relapse (B5). Although for several patients, the percentage of 70Q/H increased to some extent during treatment, no changes from inferior strain into the dominant strain were found (A6, B6). And the percentage of 70R HCV was found to be increased with treatment in several patients (A7, B7). Furthermore, the effects of IL28B SNP genotypes on the dynamic changes of 70R and 70Q/H were not observed in the study.

**Fig. 3:**
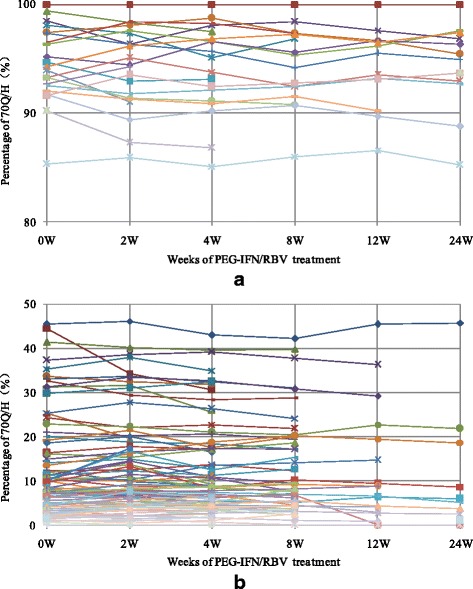
Dynamic changes in the percentage of the 70Q/H strain during treatment. **a**. Patients with 70Q/H dominance; **b**. Patients with 70R dominance

Figure 3 shows the dynamic changes in the percentage of HCV harboring the 70Q/H substitution in all 112 patients during PEG-IFNα/RBV therapy. No changes from inferior strain into the dominant strain were found. Furthermore, the samples shown in Figure 2 were subjected to TA cloning and sequencing when HCV RNAs were detectable. A total of 480 clones (20 for each time point) were picked, sequenced, and analyzed. The results showed that the percentages of 70R at each time point were comparable to the ratios of 70R to 70Q/H determined by cloning sequencing. No positive selection effect was observed for either 70Q/H or 70R (Table 3).

**Table 3:** Ratio of 70R to 70Q/H during treatment determined by cloning and sequencing

Patients	0 W		2 W		4 W		8 W		12 W		24 W	
	70R	Codon 70	70R	Codon 70	70R	Codon 70	70R	Codon 70	70R	Codon 70	70R	Codon 70
A1/B1	81.3 %	16CGG/4CAG	79.9 %	15CGG/4CAG/1 N.D.	83.4 %	16CGG/2CAG/2 N.D.	/	/	/	/	/	/
A2/B2	97.6 %	14CGA/5CGG/1CAG	97.8 %	13CGA/4CGG/3 N.D.	97.2 %	14CGA/5CGG/1 N.D.	96.8 %	14CGA/4CGG/2 N.D.	97.3 %	15CGA/5CGG	97.4 %	15CGA/3CGG/1CAG/1 N.D.
A3/B3	3.7 %	18CAG/2 N.D.	2.4 %	17CAG/1CGT/2 N.D.	3.4 %	20CAG	4.7 %	19CAG/1CGT	3.8 %	18CAG/1CGT/1 N.D.	2.4 %	19CAG/1 N.D.
A4/B4	9.7 %	2CGG/18CAA	/	/	/	/	/	/	/	/	6.2 %	1CGG/18CAA/1 N.D.
A5/B5	98.5 %	4CGG/14CGA/1CAG/1 N.D.	/	/	/	/	/	/	/	/	99.0 %	4CGG/13CGA/3 N.D.
A6/B6	89.9 %	18CGG/2CAG	82.5 %	17CGG/3CAG	/	/	/	/	/	/	/	/
A7/B7	55.5 %	11CGG/8CAG/1 N.D.	65.6 %	13CGG/7CAG	69.4 %	13CGG/6CAG/1 N.D.	/	/	/	/	/	/

N.D., no data available due to sequencing failure

## Discussion

HCV infection is a worldwide healthcare problem, but its prevalence differs among geographic regions [22]. In China, although hepatitis B virus (HBV) infection is the main cause of viral hepatitis, HCV infection is also a serious problem [23, 24]. According to a sero-epidemiological study on hepatitis C in China carried out in 2006, the overall prevalence of anti-HCV was 0.43 % (95 % CI: 0.33–0.53 %) among the population aged 1 year to 59 years of age [25]. Thus, the prevalence of HCV infection in China is low. However, given China’s population base of 1.34 billion, the number of HCV-infected patients is estimated to be nearly 40 million, and approximately 60 % of HCV patients are infected with genotype 1b. Because safer and more effective DAAs are still not available in China, PEG-IFNα/RBV combination therapy is still the standard treatment for these patients.

Several factors have been associated with the failure to respond to PEG-IFNα/RBV therapy and the incidence of post-treatment relapse. Of these, substitution of amino acid 70 in the core protein is one of the most important factors [15–19]. Several studies [26–28] have shown that the substitution of R70Q/H is more common in non-virological responders (NVRs), and that decreases in the levels of HCV RNA during treatment were significantly less in patients with 70Q/H than in patients with 70R. Amino acid substitution of R70Q/H in patients with HCV 1b had a significant effect on combination therapy in NVRs. However, most of these data were derived from studies performed in Japan, and the exact mechanisms of this association remain unclear. For example, it remains to be determined whether substitution of R70Q/H is still useful in predicting virological responses in Chinese patients. Differences in the response of 70Q/H strains to treatment compared to that of 70R strains also remain to be characterized, as does whether these mutant strains are independently resistant to treatment. Finally, whether substitution of aa70 is due to selection pressure resulting from PEG-IFNα/RBV treatment remains to be determined.

Previously, we developed a TaqMan two step real time RT-PCR system using degenerate TaqMan-MGB probes with inosine to quantify the genotypes of HCV aa70. The degenerate probes could detect 1 % of 70R or 70Q/H in the mixture with a detection limit of 10^3^ copies/mL. Cross-reactivity tests confirmed the specificity of this assay, and further cloning and sequencing analyses confirmed the reliability of the system. Using this quantitative system, the analysis of serial serum samples from 112 Chinese patients with chronic HCV 1b infection who received PEG-IFNα/RBV therapy brought us surprising and interesting results. Contrary to our original hypothesis, most patients were infected with mixtures of 70R and 70Q/H strains, and the 70R strain was dominant (79.5 % of patients) before treatment.

Among all the 104 patients who completed the 48 weeks of combination therapy and 24 consecutive weeks of follow-up, 69.2 % (72/104) of the patients achieved SVR, which is relatively higher than previously reported in Japan, Europe and the United States. The reason may be that the frequency of favourable genotype (CC) of IL28B SNPs (rs12979860) in Chinese patients is much higher than that in Japanese or Aframerican and more likely to achieve a SVR. In this study, 81.7 % of the patients were found to be the favourable genotype (CC) and 77.7 % of them achieved SVR.

Although 35.0 % of patients with dominant 70Q/H achieved SVR, while 77.4 % with dominant 70R exhibited SVR, the viral kinetics of 70R and 70Q/H always changed synchronously during treatment, no matter which strain was dominant or which genotype was the IL28B SNP. The 70Q/H strain was not independently resistant to PEG-IFNα/RBV treatment. If patients responded to treatment, the viral loads of 70Q/H decreased as well as those of 70R, while if the patients failed to respond, both the 70R and 70Q/H strains resisted treatment.

However, this finding does not mean that aa70 substitutions have no effects on the action of IFNα. Previous studies [29–32] reported that HCV core protein might be associated with resistance to IFN therapy involving the Jak-STAT signaling pathway. A study [33] also reported that aa substitutions in the core region might affect some proteins involved in resistance to IFNα therapy, such as SOCS proteins, which is known to inhibit IFNα-induced activation of the Jak-STAT pathway and expression of the antiviral proteins 2′,5′-OAS and MxA. Further study [34] showed that HCV R70 core mutants were resistant to IFN in vitro, and the resistance may be induced by IL-6-induced upregulation of SOCS3. These mechanisms can explain clinical IFNα resistance in patients with HCV core mutants, but are not consistent with our data showing that core 70R and core 70Q/R respond similarly during treatment. Unlike DAAs, which inhibit the replication of HCV directly, IFNα exerts antiviral effects by inducing the expression of antiviral proteins. Presumably, if 70R and 70Q/H exist in a single hepatic cell, the both 70R and 70Q/H core proteins will be expressed. Only when the 70Q/H core protein is dominant, the expression of antiviral proteins induced by interferon will be down-regulated significantly, and thus influence the inhibition of not only 70Q/H, but also the 70R strains. However, this has yet to be confirmed. An alternative explanation is that R70Q/H substitution alone is not sufficient to produce resistance to IFNα. Ikeda et al. [35] designed a study to examine the differences among the antiviral activities of HCV core proteins with various substitutions at aa70 and/or aa91 in vitro. Retroviral vectors expressing the HCV core proteins with substitutions of arginine/leucine, arginine/methionine, glutamine/leucine or glutamine/methionine at aa70/aa91 were transiently transfected or stably transduced into an immortalized hepatocyte line (PH5CH8), hepatoma cell lines, and an HCV-RNA replicating cell line (sOR) to evaluate the antiviral responses to IFN-α or IFN-α/RBV. The results showed that the promoter activity levels of IFN-stimulated genes in the transiently transfected cells or the mRNA levels of 2′-5′-oligoadenylate synthetase in the stably transduced PH5CH8 cells were not associated with HCV core aa70 and/or aa91 substitutions during IFNα treatment. Antiviral responses to IFNα or IFNα/RBV treatment were enhanced in sOR cells stably transduced with the HCV core, although there were no differences in antiviral responses among the cells expressing different core types. Furthermore, Hiraga N, et al.[36] found that core aa70 substitutions did not impaired the infectivity and replication ability of infectious HCV genotype 1b clone HCV-KT9 in human hepatocyte chimeric mice, and the effect of IFN treatment was similar in wild-type and mutant viruses. These results were in agreement with those of our clinical studies. Therefore, the detailed mechanism should be investigated further to clarify the discrepancy between these studies.

In addition, dynamic changes in the proportions of 70R and 70Q/H strains during treatment showed that the ratios of 70Q/H to 70R did not change significantly. Although for several patients, the percentage of 70Q/H increased to a small extent, no changes from inferior strain into the dominant strain were found. As a result, no evidence of positive selection for the core 70Q/H variant induced by PEG-IFNα/RVB treatment was observed in the present study. This disagrees with a previous report investigating the viral factors associated with treatment failure determined by direct and cloning sequencing that indicated that treatment-induced selection occurred in all nonresponsive patients who harbored 70Q quasispecies detectable by cloning [37]. The discrepancy between these two studies may be associated with the detection methods, ethnic and genetic differences, or some unexplained mechanism.

## Conclusions

Most Chinese patients with HCV 1b examined in this study were infected with a mixture of 70R and 70Q/H strains before treatment. Assessment of the dynamic changes in the proportion of 70R and 70Q/H strains during PEG-IFNα/RBV therapy showed that 70Q/H had similar response to PEG-IFN/RBV therapy as 70R and indicated that substitution of R70Q/H is not enough to lead to resistance to IFNα. No evidence of positive selection for 70Q/H induced by treatment with PEG-IFNα/RBV was observed. Furthermore, IL28B SNP genotypes were not associated with the dynamic changes of 70R and 70Q/H strains during PEG-IFNα/RBV therapy. However, the ratio of 70Q/H to 70R and IL28B polymorphism might have an effect on virological response to treatment. Further studies are required to investigate the detailed mechanism of this association.

## Methods

### Ethics statement

The study protocol conformed to the ethical guidelines of the Declaration of Helsinki and was approved by The Ethical Committee of Beijing Youan Hospital, Capital Medical University. Written informed consent was obtained from each patient participating in this study.

### Patient population

Between January 2009 and June 2013, 112 Chinese patients with chronic HCV 1b infection were enrolled in this study at Beijing Youan Hospital, Capital Medical University. Patients were prospectively selected based on the following criteria: (1) levels of HCV RNA greater than 1 × 10^5^ IU/mL as measured with TaqMan real time PCR (Amplicor, Roche Diagnostic Systems, Shanghai, China); (2) naive to antiviral treatment; (3) free of coinfection with hepatitis B virus or human immunodeficiency virus; (4) free of HCC based on laboratory tests and imaging studies; and (5) free of other causes of liver disease, such as alcoholic liver disease, autoimmune liver disease, drug induced liver injury, hemochromatosis, or Wilson’s disease.

All patients enrolled in this study received a standard protocol of 48 weeks of combination therapy with PEG-IFNα-2b (1.5 mg/kg of body weight by subcutaneous injection once per week) plus RBV (600–1200 mg daily, according to body weight) and 24 consecutive weeks of follow-up. All patients underwent HCV RNA testing at weeks 4, 8, 12, 24, and 48 of therapy. Follow-up testing was performed at week 72. When needed, doses of PEG-IFNα-2b and RBV were reduced on an individual basis during treatment to lessen adverse effects, and these dose reductions were performed according to the guidelines for the treatment of hepatitis C in China.

### Serum samples

Blood samples were obtained at least once within the month before treatment. During combination therapy, blood samples were obtained at weeks 2, 4, 8, 12, and 24. Serum samples were frozen at −80 °C within 4 h of collection and thawed at the time of measurement.

### Definition of virological responses

Virological responses during therapy were defined based on the results of HCV RNA analysis. Early virological response (EVR) was defined as a more than two log reduction in HCV RNA levels compared to baseline (partial EVR) or as HCV RNA negative at treatment week 12 (complete EVR). Sustained virological response (SVR) was defined as undetectable levels of HCV RNA 24 weeks after cessation of treatment. Breakthrough was defined as the reappearance of HCV RNA in serum while still on therapy. Relapse was defined as the reappearance of HCV RNA in the serum after therapy was discontinued. Non-virological response (NVR) was defined as failure to clear HCV RNA from the serum after 24 weeks of therapy.

### HCV RNA extraction and reverse transcription

HCV RNA was extracted from the serum samples (140 μL) using the QIAamp Viral RNA Mini Kit (Qiagen, Shanghai, China), and cDNA was prepared by reverse transcription with random hexamers using TaqMan Reverse Transcription Reagents (Applied Biosystems). The reaction conditions were 25 °C for 10 min, 42 °C for 40 min, and 95 °C for 5 min.

### IL28B SNP genotyping

Two IL28B SNPs, rs12979860 and rs8099917 were genotyped for each patient as previously described [38]. Briefly, human genomic DNA was extracted from peripheral blood cells by a QIAamp DNA Mini Kit (Qiagen). Genotyping of the rs12979860 and rs8099917 was performed using direct sequencing. Nucleic acids were amplified by PCR using specific primers (rs12979860: 5′-ATTCCTGGACGTGGATGGGTAC-3′ and 5′-AGCGCGGAGTGCAATTCA-3′; rs8099917: 5′-TTGTCACTGTTCCTCCTTTTGTTT-3′ and 5′-TGGGAGAATGCAAATGAGAGATA-3′). Amplicons were purified with a QIAquick PCR Purification Kit (Qiagen) after agarose gel electrophoresis and then used for direct sequencing (Beijing AuGCT DNA-SYN Biotechnology Co. Ltd).

### Quantification of HCV RNA with 70R and 70Q/H

Previously, we developed a TaqMan two-step real time RT-PCR system for the quantification of HCV RNA with 70R and 70Q/H using degenerate TaqMan-minor groove binder (MGB) probes with inosine [21]. According to the proportions of codon 70 types in the HCV 1b gene from genetic databases [39], the degenerate probes can be used to detect 99.6 % of patients with HCV 1b. Using this method, 70R and 70Q/H viral RNAs can be quantified and their dynamic responses to PEG-IFNα/RBV therapy assessed.

The detection system used is as follows. Based on the consensus sequence of the HCV 1b core gene identified previously [39], a primer pair was designed based on the conserved regions. Meanwhile, two degenerate TaqMan-MGB probes with inosine (I) were designed to distinguish between the 70R (CGN) and 70Q/H (CAN) codons. Real time PCR was performed in a final volume of 50 μL containing 5 μL of the cDNA reaction mixture, 0.3 μM of each primer, 0.1 μM probes, and 25 μL 2 × LightCycler® 480 Probes Master Mix (Roche Applied Science, Shanghai, China). Two separate reaction systems were prepared to detect 70R and 70Q/H, respectively, but the two reactions were carried out simultaneously on the same real time PCR system. The cycle conditions were as follows: an initial denaturation for 10 min at 95 °C, followed by 45 cycles of denaturation for 15 s at 95 °C, and an annealing/extension step for 1 min at 60 °C. All reactions were performed in triplicate on a LightCycler® 480 Real-Time PCR System (Roche Applied Science) and the results were analyzed using LightCycler® 480 Software (Roche Applied Science).

### Nucleotide sequencing of the HCV core gene

The ratio of 70R and 70Q/H was confirmed by cloning and sequencing some of the patients. Cloning was carried out using TOPO TA Cloning Kits (Invitrogen), according to the manufacturer’s protocol. PCR was performed using 5′-TCGTGGAAGGCGACAACC-3′ and 5′-GCCGACGAGCGGAATGT-3′ as the sense and antisense primers, respectively. A total of 20 colonies for each sample were selected at random and sequenced (Beijing AuGCT DNA-SYN Biotechnology Co., Ltd).

### Statistical Analysis

The one-sample Kolmogorov-Smirnov test was used to test the normality of the baseline clinical data. The relationship between the dominant strain and the virological response was analyzed by Chi-square tests and Fisher’s exact probability test with a 4-fold table. A *P*-value of <0.05 was considered statistically significant. Statistical analyses were performed using SPSS software (SPSS Inc., Chicago, IL).

## Abbreviations

aa: amino acid
ALT: alanine aminotransferase
AST: aspartate aminotransferase
DAAs: direct-acting antivirals
EVR: early virological response
HBV: hepatitis B virus
HCC: hepatocellular carcinoma
HCV: hepatitis C virus
IL28B SNPs: interleukin 28B single nucleotide polymorphisms
MGB: minor groove binder
NVR: non-virological response
PEG-IFN: pegylated interferon
PT: prothrombin time
RBV: ribavirin
SVR: sustained virological response
